# Effects of Physical Activity Interventions on Cognitive Flexibility in Children with ADHD: A Systematic Review

**DOI:** 10.3390/sports14090389

**Published:** 2026-09-02

**Authors:** Carlos Alberto Lubián Rodríguez, Alexandra Martín-Rodríguez

**Affiliations:** 1 Faculty of Sports Sciences, Universidad Europea de Madrid, Tajo Street, s/n, 28670 Madrid, Spain; carlos2002862@gmail.com; 2 Faculty of Health Sciences, UNIE Universidad, Av. de Monforte de Lemos, 28, Fuencarral-El Pardo, 28029 Madrid, Spain

**Keywords:** attention deficit hyperactivity disorder, cognitive flexibility, executive function, physical activity, exercise, children, systematic review

## Abstract

Background: Attention Deficit Hyperactivity Disorder (ADHD) is associated with impairments in executive functions, particularly cognitive flexibility, which may negatively affect academic performance, behavioral regulation, and daily adaptation in children. Physical activity has emerged as a promising non-pharmacological strategy to support executive functioning; however, evidence regarding the effects of different exercise modalities on cognitive flexibility remains heterogeneous. Methods: This systematic review followed the Preferred Reporting Items for Systematic Reviews and Meta-Analyses (PRISMA) guidelines. The review protocol was retrospectively registered in the PROSPERO database (CRD420261399699) before manuscript submission. Searches were conducted in PubMed, Scopus, MEDLINE, Web of Science, PsycInfo, and SPORTDiscus. Studies examining the effects of physical activity interventions on cognitive flexibility in Primary Education students with ADHD were included. Seventeen studies involving 634 participants aged 6–12 years met the eligibility criteria. Methodological quality was assessed using design-appropriate critical appraisal tools. Results: Overall, the included studies reported potentially beneficial effects of physical activity on cognitive flexibility, although findings varied according to study design, intervention characteristics, and outcome measures. Across the available studies, multi-session interventions tended to report more consistent improvements across cognitive flexibility outcomes than single-session protocols. Structured sports and interventions combining physical and cognitive demands, including football, table tennis, EXCIR, and BARAN, also reported improvements in several cognitive flexibility outcomes. However, substantial methodological heterogeneity and the absence of direct comparisons between intervention types or durations preclude conclusions regarding the superiority or comparative effectiveness of specific approaches. Conclusions: Physical activity may represent a promising complementary strategy for supporting cognitive flexibility in children with ADHD. The available evidence suggests potentially favorable findings for multi-session and cognitively engaging interventions; however, these patterns should be interpreted cautiously because of the heterogeneity of study designs, intervention protocols, and cognitive flexibility measures. Further well-designed studies directly comparing intervention characteristics and exercise modalities are needed before specific recommendations regarding optimal type or duration can be established.

## 1. Introduction

Attention Deficit Hyperactivity Disorder is defined as one of the most prevalent neurodevelopmental disorders in the child and adolescent population worldwide, with an estimated rate ranging between 5.29% and 8% [1,2]. This condition is fundamentally characterized by levels of inattention, hyperactivity, and impulsivity that are inappropriate for the individual’s level of development [3].

These symptoms can be classified into two types of behavioral problems. Lack of attention is one of the most characteristic symptoms of ADHD and is indispensable for its diagnosis. Students present difficulties in concentration, persistence, and the ability to focus attention, showing vulnerability to external stimuli. Hyperactivity and impulsivity constitute another central symptom of the disorder, reflected in a level of activity considerably higher than that of students with typical development, especially in situations that require remaining seated or following an order [3,4], thus affecting behavior, emotion, and executive functions in these types of students [5]. In the context of Primary Education, these clinical manifestations not only affect sustained attention, self-regulation, and daily adaptive functioning of students [6,7], but also frequently lead to multidimensional difficulties that impact academic competencies, emotional regulation, and social interaction skills [8,9].

From a neuropsychological perspective, deficits in executive functions are considered a distinctive characteristic in students with ADHD [4]. Executive functions are understood as higher-order cognitive processes essential for self-regulation and the achievement of goal-directed behaviors [10]. Within their basic structure, three main components are identified: inhibitory control, working memory, and cognitive flexibility [11]. This last one, the central object of the present review, is mainly divided into two subcategories: reactive cognitive flexibility, referring to the ability of an individual to adjust response strategies in the face of changes in environmental demands or task rules, and spontaneous cognitive flexibility, referring to the ability to generate by oneself a wide variety of new ideas, without external pressures to make changes, to explore the unknown, and to adjust one’s thinking [12,13]. A deficient development of these capacities results in behavioral rigidity and inefficiency in problem solving, seriously compromising students’ academic performance and daily adaptation [14,15].

The treatment of ADHD has mainly focused on the use of drugs and behavioral psychological interventions [16,17]. Although pharmacological treatments are effective for many children with ADHD, individual responses vary, and their use may be accompanied by adverse effects and tolerability concerns. These limitations have contributed to continued interest in complementary non-pharmacological interventions [18,19,20]. Considering this, physical activity plays an important role, as it has emerged as a therapeutic strategy with great potential due to its feasibility, low cost, and absence of harmful side effects [18,21]. Current scientific evidence suggests that physical activity acts as a powerful endogenous stimulus capable of triggering neuroplasticity processes and structural changes in critical brain regions, such as the prefrontal cortex and the basal ganglia [22,23,24]. At the same time, physical exercise has been proposed as an enhancer of cerebral blood flow and the release of essential neurotransmitters such as dopamine and norepinephrine, whose regulation is altered in the brain of students with ADHD [25,26,27]. Likewise, physical activity stimulates the production of brain-derived neurotrophic factor (BDNF), which plays a regulatory role in the plasticity of neuronal structures and functions, facilitating the efficiency of the neural circuits that support cognitive flexibility, among other executive functions [28,29].

**Figure 1:**
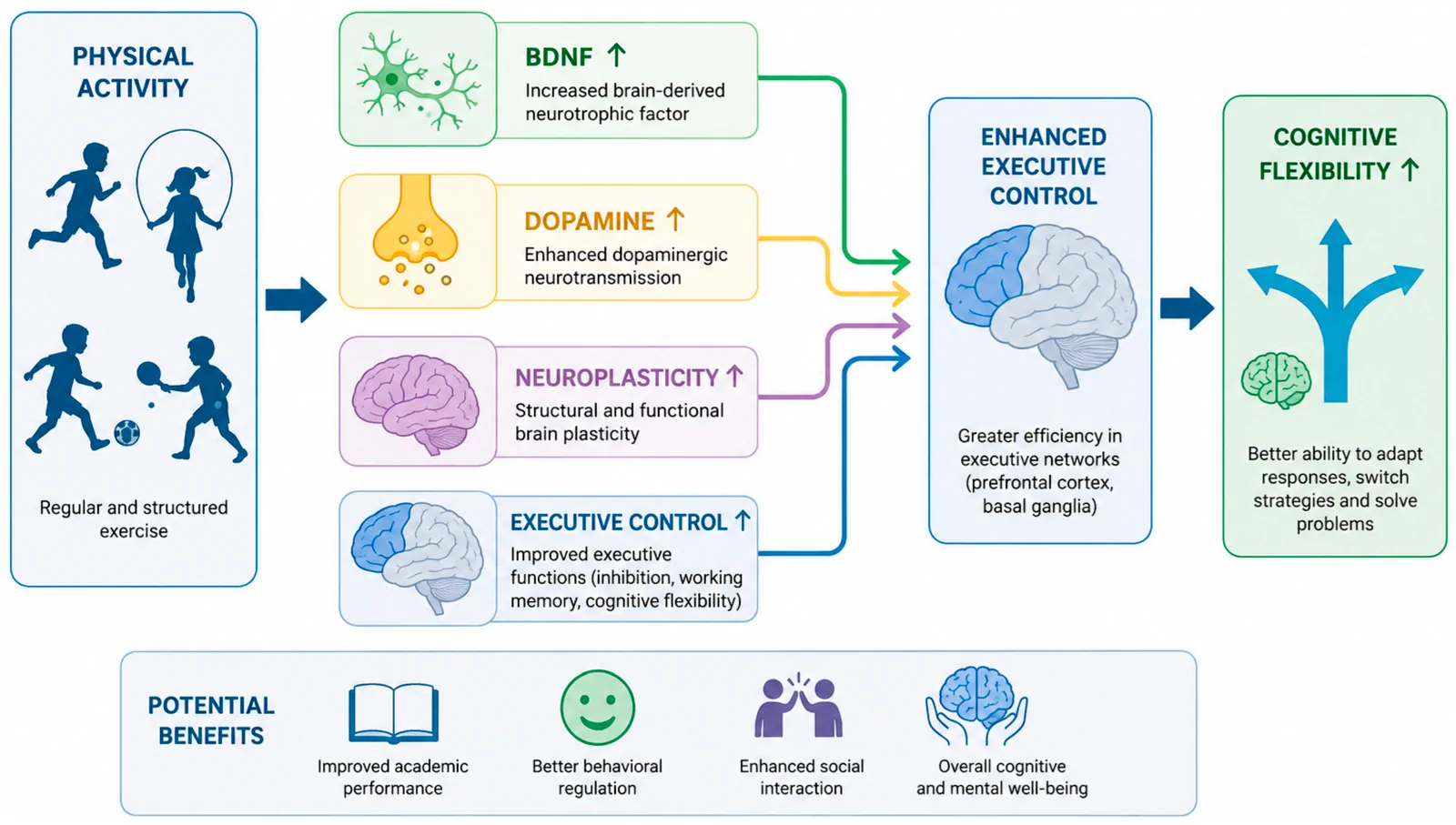
Potential neurobiological and cognitive mechanisms linking physical activity and cognitive flexibility in children with ADHD.

Even so, the effects of physical activity on cognitive flexibility appear to be modulated by various variables: exercise intensity, program duration, or the type of cognitive demand of the exercise. Moderate-intensity aerobic exercise has shown consistent benefits [30]. On the other hand, other modalities such as activities that require reactions in dynamic and unpredictable environments or exergaming could offer additional advantages by combining motor challenge with a high demand for information processing (Figure 1).

Although there is an increasing amount of scientific literature, a notable heterogeneity in the results and a lack of consensus persist regarding which specific protocols are the most effective for enhancing cognitive flexibility in a stable manner in students aged 6 to 12 years with ADHD. Many previous studies have analyzed executive functions in a global way or have included samples of very diverse ages, which makes it difficult to prescribe physical activity adapted to the maturational needs of Primary Education students [4,31,32]. Although previous reviews have examined executive functions globally in ADHD populations, few studies have specifically focused on cognitive flexibility in Primary Education students, despite its relevance for academic adaptation and behavioral regulation.

For all these reasons, the objective of this systematic review was to analyze the impact of physical activity interventions on cognitive flexibility in Primary Education students with ADHD. The present research aims to provide an updated overview that serves as a guide both for health professionals and for Physical Education teachers in the design of effective and scientifically grounded intervention programs for improving the quality of life and the holistic development of students with ADHD.

## 2. Materials and Methods

This systematic review was conducted in accordance with the Preferred Reporting Items for Systematic Reviews and Meta-Analyses (PRISMA) guidelines. The review protocol was retrospectively registered in the PROSPERO database (Registration No. CRD420261399699). The review was originally conducted as part of a Master’s thesis, and registration was completed before manuscript submission to improve transparency and facilitate dissemination of the work [33]. Minor differences exist between the retrospectively registered PROSPERO record and the final review methodology. These include the expansion of the database search, clarification of the publication period, and correction of the intervention description. These refinements were introduced to improve the completeness and accuracy of the review and did not modify the review question, eligibility criteria, or interpretation of the findings.

### 2.1. Search Strategy

A systematic literature search was conducted in the electronic databases PubMed, MEDLINE, Scopus, Web of Science, PsycInfo, and SPORTDiscus to identify studies investigating the effects of physical activity interventions on cognitive flexibility in Primary Education students diagnosed with Attention Deficit Hyperactivity Disorder (ADHD).

The search strategy combined controlled vocabulary terms (MeSH) and free-text keywords related to four main domains: (1) ADHD, (2) physical activity and exercise, (3) cognitive flexibility and executive functions, and (4) children and school-age populations. Boolean operators (“AND”, “OR”) were used to appropriately combine the search terms. The main search string was as follows: (“ADHD” OR “attention deficit hyperactivity disorder”) AND (“physical activity” OR exercise OR sport OR exergaming OR aerobic exercise) AND (“cognitive flexibility” OR “executive function” OR “task switching” OR “set shifting”) AND (children OR schoolchildren OR “primary education”).

The search covered studies published between 2011 and February 2026, corresponding to the publication period of the 17 studies ultimately included in this review. Most of the included studies were randomized controlled trials (RCTs; *n* = 14), while two studies used a within-subject design and one was a case report. During the screening process, studies were excluded if they did not include a physical activity intervention, were not written in English or Spanish, were systematic reviews or meta-analyses, or were not original research articles.

Additionally, the reference lists of the included studies and previous systematic reviews were manually screened to identify potentially relevant articles not retrieved through the electronic database search. The final search was conducted on 23 February 2026, and all references were imported into Zotero software (version 7.0.16) for duplicate removal and study management.

### 2.2. Inclusion and Exclusion Criteria

The inclusion items were established based on the structured review question using the PICO (Population, Intervention, Comparison and Outcome) model [34]. The items were as follows: (1) participants were students aged 6 to 12 years diagnosed with ADHD; (2) studies that carried out a physical activity intervention on cognitive flexibility were included; (3) studies that assessed cognitive changes after the intervention were considered; (4) assessment of cognitive flexibility using interval or ratio scale measures.

The studies excluded from the review were those that met any of the following characteristics: (1) no physical activity intervention was performed; (2) they were not written in English or Spanish; (3) systematic reviews or meta-analyses; (4) they were not original studies.

Medication use was not established as an inclusion or exclusion criterion because the review aimed to represent the clinical population of children with ADHD, in whom pharmacological treatment is common. However, medication status and its management during the intervention and cognitive assessments were considered potential confounding factors and were extracted whenever reported by the primary studies.

### 2.3. Study Selection

The studies identified in the different databases were imported into Zotero software (7.0.16). Subsequently, duplicates were manually removed. Then, the selection was carried out by reading titles and abstracts. Finally, the full texts were read to decide their final inclusion or exclusion.

### 2.4. Data Extraction

Demographic data, study characteristics, intervention characteristics, and results related to cognitive flexibility were extracted.

The demographic information collected included: first author and year of publication, country where the study was conducted, sample size, sex, and mean age of participants. Regarding the intervention, the type of physical activity, session intensity, duration of each session, weekly frequency, total duration of the intervention, and instruments used to assess cognitive flexibility were extracted. Finally, scores from cognitive flexibility tasks and their correlations with different variables were also extracted. Information regarding ADHD medication was also extracted whenever available, including medication status, type of treatment, stability of dosage, continuation or temporary withdrawal during the intervention, and timing relative to cognitive assessment. Because these data were inconsistently reported and managed across the included studies, it could not be incorporated as a covariate or used for subgroup comparisons and was therefore considered qualitatively in the narrative synthesis.

All this data was organized into comparative tables. Data extraction was performed independently by two reviewers using a predefined data extraction form. Extracted information includes study characteristics, participant details, intervention characteristics, outcome measures, and results related to cognitive flexibility. Any discrepancies between reviewers were resolved through discussion and, if necessary, consultation with a third reviewer.

Two reviewers independently screened all titles and abstracts retrieved from the database search. Subsequently, full-text articles were independently assessed for eligibility according to the predefined inclusion and exclusion criteria. Any disagreements were resolved through discussion until consensus was reached. When necessary, a third reviewer was consulted to resolve discrepancies. Although inter-rater agreement statistics (e.g., Cohen’s kappa) were not calculated, the independent screening process aimed to minimize selection bias.

Due to the substantial methodological heterogeneity among the included studies, a quantitative meta-analysis was not considered appropriate. Specifically, considerable variability was observed in the types of physical activity interventions (e.g., aerobic exercise, exergaming, football, swimming, table tennis, EXCIR, BARAN, mindfulness-based exercise, and neurocognitive training), intervention characteristics (duration, frequency, and intensity), study designs (randomized controlled trials, within-subject studies, and a case report), and the instruments used to assess cognitive flexibility (e.g., WCST, TMT, Flanker Task, Stroop Test, Task Switching Paradigm, and E-Prime 2.0). Furthermore, only a small number of studies were available for each specific intervention modality, limiting the feasibility of meaningful quantitative pooling. Therefore, a narrative synthesis was conducted to provide a comprehensive and accurate interpretation of the available evidence. Accordingly, consistent with PRISMA 2020 recommendations (Table S1), the findings were synthesized narratively rather than quantitatively.

### 2.5. Methodological Quality and Critical Appraisal

The Physiotherapy Evidence Database (PEDro) scale was used to assess the methodological quality of the randomized controlled trials (RCTs) included in this study. This instrument has shown acceptable reliability for evaluating methodological quality in physical activity studies. The PEDro scale includes 11 items: (1) eligibility criteria; (2) random allocation; (3) allocation concealment; (4) baseline comparability of groups; (5) blinding of participants; (6) blinding of therapists or instructors; (7) blinding of assessors; (8) adequate follow-up; (9) intention-to-treat analysis; (10) between-group comparisons; (11) measures of precision and variability. Scores range from 0 to 10. The first item is not scored, as it assesses external validity of the study rather than internal validity. Each fulfilled item adds one point; non-fulfilled or unreported items receive zero points. Study quality was classified as: 9–10 excellent; 6–8 good; 4–5 acceptable; <4 low [35].

On the other hand, for non-randomized studies and within-subject designs, the Joanna Briggs Institute (JBI) Critical Appraisal Tools were used. This instrument includes 9 items: (1) clarity of the causal relationship; (2) baseline comparability of groups; (3) equivalence of interventions; (4) presence of a control group; (5) pre- and post-intervention measurement; (6) complete follow-up and handling of losses; (7) consistency in outcome measurement between groups; (8) validity and reliability of outcome measures; (9) appropriateness of statistical analysis. Responses can be “yes”, “no”, “unclear”, or “not applicable” [36].

Finally, for case studies the CARE (Case Report Guidelines) guide was used. This tool includes 13 items: (1) title; (2) keywords; (3) abstract; (4) introduction; (5) patient information; (6) clinical findings; (7) timeline; (8) diagnostic assessment; (9) therapeutic intervention; (10) follow-up and outcomes; (11) discussion; (12) patient perspective; (13) informed consent. Each item is assessed as “yes” or “no” [37].

## 3. Results

**Figure 2:**
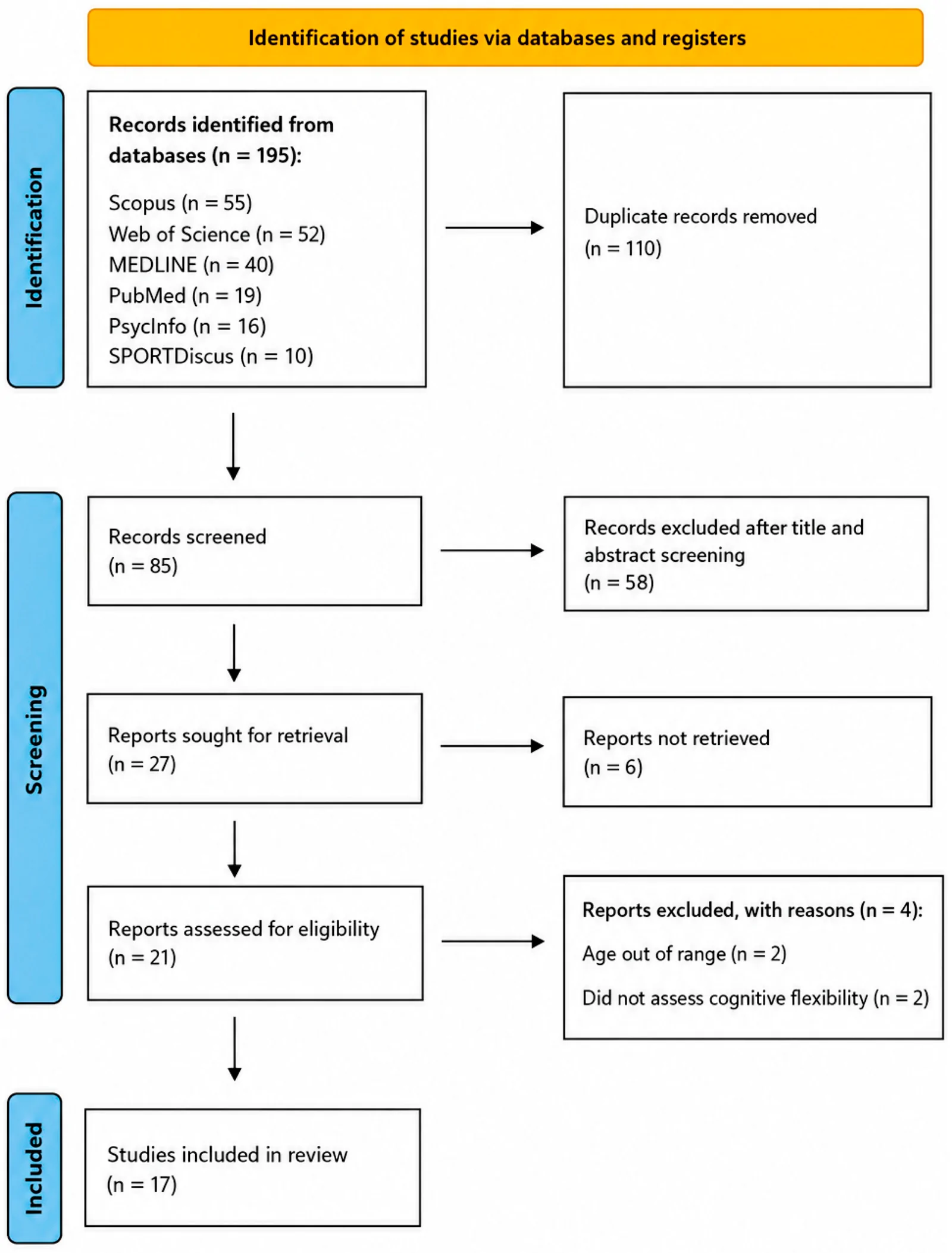
Flow diagram of the search and selection of studies.

The initial search retrieved 195 articles. After the removal of duplicates, 85 remained, of which 58 were excluded after title and abstract screening. A total of 27 articles were sought for full-text retrieval, with 6 of them not being available. Finally, 17 studies were included in this work after exhaustive review. The complete flow diagram of the selection process is shown in Figure 2 and The PRISMA information is provided in the Supplementary Material.

The reporting and management of ADHD medication varied considerably across the included studies. Some studies included participants receiving psychostimulant treatment and maintained their usual medication during the study period, whereas others required temporary medication withdrawal before cognitive assessment. In several studies, medication status, dosage, or the timing of medication relative to the intervention and outcome assessment was not reported in sufficient detail. Moreover, medication status was not consistently included in adjusted analyses or examined through subgroup comparisons. This heterogeneity prevented a separate evaluation of the effects of physical activity according to pharmacological treatment status.

### 3.1. Characteristics of the Studies

Table 1 shows relevant information on the 17 studies analyzed in this review. These were conducted in 9 different countries: the USA, Switzerland, Canada, Peru, Taiwan, Brazil, China, South Korea, and Iran. The total sample size was 634 participants (535 boys and 99 girls) and the mean age was 9.2 years. There was a total of 14 RCTs [14,38,39,40,41,42,43,44,45,46,47,48,49,50], 2 within-subject studies [51,52] and 1 case report [53]. Turning to the intervention, it lasted between 4 and 12 weeks in 11 studies [40,41,43,44,45,46,47,48,49,50,53], while in 6 studies it lasted a single week [14,38,39,42,51,52]. Regarding frequency, the interventions were usually applied between 3 and 5 times per week for 30–90 min. The intensity was moderate in 4 studies [14,42,51,52], moderate to vigorous in 4 studies [39,40,45,50], and moderate to high in 1 study [46], and 8 studies did not report the intensity [38,41,43,44,47,48,49,53]. The types of physical activity or exercises used were aerobic exercise [14,42,44,45,50,51,52]; exergaming, which consists of performing physical activity through interactive video games that require body movement to play [39,40]; body rhythmic sequences [53]; BARAN, which is an intervention program based on balance, sensory integration, and postural control [47]; swimming [41]; football [43]; table tennis [49]; neurocognitive exercise [45,50]; EXCIR, defined as a physical activity program with progressive cognitive demand [48]; and finally, mindfulness meditation [51] and goal-oriented exercise [44,46]. The measurement instruments used to assess cognitive flexibility were the Wisconsin Card Sorting Test (WCST) [14,38,47,48,49,53], Flanker Task [39,40], Trail Making Test (TMT) [41,44,45,50,51,52], Task Switching Paradigm [42], E-Prime 2.0 software [43] and Stroop Test [46].

### 3.2. Methodological Quality

Table 2 presents the critical appraisal of the studies included in this review. The PEDro scale was used to assess the methodological quality of 14 studies. One study obtained a score < 4, indicating low quality [42]; eight studies obtained scores between 4 and 5, indicating acceptable quality [41,43,44,45,46,47,49,50]; and five studies obtained scores between 6 and 8, indicating good quality [14,38,39,40,48]. The overall mean PEDro score was 5.2, corresponding to acceptable methodological quality. The JBI Critical Appraisal Tool was applied to two within-subject studies [51,52]. Given the characteristics of these study designs, the item-level judgments were interpreted descriptively rather than converted into an overall methodological quality category. Finally, the CARE guidelines were used to assess the reporting completeness of the case report [53], rather than as a formal methodological quality or risk-of-bias scoring tool. Accordingly, the CARE results were interpreted descriptively, and no overall methodological quality classification was assigned based on the number of items fulfilled.

### 3.3. Cognitive Flexibility

The studies included in this review analyzed the effect of different types of physical activity on cognitive flexibility in Primary Education students with ADHD using various assessment instruments. Generally, the results showed that physical activity can generate improvements, although the effects depend on factors such as the type of exercise or the duration of the intervention. These results can be observed in detail in Table 3.

#### 3.3.1. Aerobic Exercise

Aerobic exercise is the most studied type of intervention, showing heterogeneous results depending on the duration and design of the intervention. Some studies of acute interventions observed partial improvements. A session of moderate-intensity aerobic exercise produced significant improvements in non-perseverative errors and number of categories completed in the WCST, thus indicating greater efficiency in problem solving. On the other hand, other variables such as perseverative errors, perseverative responses, or total correct responses also improved in the control group, indicating a lack of specificity of the exercise effect [14]. Similarly, other studies did not find significant improvements in cognitive flexibility after a single session, assessed through the Task Switching Paradigm and the TMT Part B, respectively, demonstrating a low sensitivity of this capacity to acute exercise [42,52]. Turning to more prolonged interventions, aerobic exercise showed more consistent results. Programs of several weeks produced significant improvements in cognitive flexibility, especially in tasks assessed through the TMT. At the same time, it was observed that part of these improvements was mediated by changes in sleep variables, such as the reduction in sleep latency, associated with better sports performance [45,50]. Not all aerobic interventions showed positive effects. One study that compared aerobic exercise with mindfulness meditation found that only the latter intervention produced improvements in cognitive flexibility; these were temporary and the intervention lasted one session [51].

#### 3.3.2. Exergaming

Interventions based on exergaming showed relatively consistent results in improving cognitive flexibility, especially in variables related to processing speed. In this case, both in acute interventions and in programs lasting several weeks, significant reductions in reaction times were observed in tasks assessed through the Flanker Task, particularly in switch trials and in global switch costs, although there were no improvements in task accuracy [39,40]. Furthermore, it was observed that cognitive flexibility assessed through reaction times shows a significant relationship with other executive functions. Overall, exergaming appears to be an effective intervention for improving processing speed associated with cognitive flexibility, although its impact on accuracy and other more complex components is limited.

#### 3.3.3. Specific Sports and Activities

Intervention programs based on specific sports and structured activities have shown positive and consistent results in improving this executive function. A football program carried out over 6 weeks produced significant improvements compared to usual school activities, highlighting that the effects depended on the interaction between intervention time and group membership of the students [43]. Likewise, the practice of table tennis generated significant improvements in multiple indicators of the WCST: total correct responses, perseverative errors, and number of categories completed [49]. At the same time, an 8-week aquatic exercise program showed significant improvements in cognitive flexibility in students with ADHD [41]. On the other hand, a study based on an intervention using body rhythmic sequences did not show significant changes in this capacity [53].

In general, sports and structured physical activities seem to enhance cognitive flexibility, especially when they involve decision-making, adaptation to stimuli, and motor variability.

#### 3.3.4. Interventions with Cognitive Demand

Interventions that combine physical activity with cognitive demands or dual tasks showed the positive effects on cognitive flexibility. A program of structured goal-oriented exercises produced significant improvements in multiple indicators of the Stroop Test, reaction time, number of correct responses, and errors, reflecting improvements in both speed and accuracy of cognitive processing, although without changes in interference control [46]. The BARAN and EXCIR programs showed significant improvements in key variables of the WCST: reduction in perseverance errors and increase in the number of categories completed, as well as greater efficiency in response time. These results indicate that interventions that integrate postural control and cognitive demands improve cognitive shifting ability and reduce repetitive or erroneous responses [47,48]. At the same time, a 12-session program of varied exercises with a cognitive component improved mental shifting ability: reducing execution time and showing positive relationships with inattention variables and social skills [44].

In general, this type of intervention showed relatively consistent positive findings across the included studies; however, the available evidence does not permit conclusions regarding its comparative effectiveness relative to other exercise modalities.

#### 3.3.5. Other Experimental Conditions

One study compared different experimental conditions (walking, standing, and sitting). No statistically significant differences in cognitive flexibility were found among the conditions. Although the standing group showed numerically greater improvements than the walking and sitting groups, these differences should be interpreted as a descriptive trend rather than evidence of an intervention effect [38].

In summary, the results suggest that physical activity can improve cognitive flexibility in students with ADHD, although the effectiveness varies according to the type of intervention. Activities with a higher cognitive or coordinative component, such as structured sports, seem to generate the most consistent effects. In contrast, isolated aerobic exercise, especially in short-duration interventions, shows more limited results. In addition, factors such as sleep may partially mediate the effects of exercise on this executive function.

#### 3.3.6. Chronic Intervention vs. Acute Intervention

The studies included in this systematic review allow for a descriptive examination of the findings reported for acute and chronic interventions. However, because these interventions were evaluated across heterogeneous studies rather than through direct comparisons, no conclusions regarding the superiority of one intervention duration over another can be established. In general, single-session interventions show heterogeneous and limited results. Some studies report isolated improvements, mainly in variables related to processing speed, such as reduced reaction times or specific improvements in indicators of the WCST: non-perseverative errors or completed categories. These effects were not consistent across all variables or all studies, with several cases showing no significant differences compared to control groups. At the same time, when improvements were detected, these were temporary or partial, affecting more specific components rather than cognitive flexibility as a whole [14,38,39,40,42,52]. Across the included studies, multi-session interventions were associated with more consistently reported improvements across several cognitive flexibility outcomes. Nevertheless, this pattern should be interpreted cautiously because of differences in study design, intervention characteristics, outcome measures, and methodological quality. These programs produced significant improvements in multiple indicators of cognitive flexibility: reduction in perseverative errors, increase in completed categories, and improvement in cognitive processing efficiency. At the same time, they allowed for the observation of more global effects, affecting both speed and accuracy of responses, and even showing relationships with other variables such as sleep, attention, or social skills. In addition, chronic intervention programs, especially those that incorporate cognitive demands, seem to promote more stable adaptations over time, in contrast to the more immediate but less shorter-lasting nature of acute intervention [40,41,43,44,45,46,47,48,49,50,53].

## 4. Discussion

The present systematic review aimed to clarify the impact of physical activity interventions on cognitive flexibility in Primary Education students with ADHD. The findings of the 17 included studies indicate that regular physical activity constitutes an effective complementary strategy for improving cognitive flexibility, an executive function that is essential for academic performance, behavioral regulation, and problem-solving abilities. This review also provides an updated synthesis focused specifically on cognitive flexibility, an executive function that has received comparatively less attention than executive functioning as a whole.

A pattern observed across the included studies was that multi-session interventions tended to report more consistent improvements in cognitive flexibility than single-session interventions. However, given the methodological heterogeneity and absence of direct comparisons between intervention durations, this finding should not be interpreted as evidence of superiority of chronic over acute exercise. While a single session of aerobic exercise may induce immediate improvements in processing speed and certain executive processes, these effects appear to be transient. In contrast, intervention programs lasting between 4 and 12 weeks consistently produced broader and more sustained improvements in cognitive flexibility. These findings are consistent with previous systematic reviews and meta-analyses highlighting intervention duration and regular participation as key determinants of cognitive benefits [30,31,54].

Regarding exercise modality, the present findings suggest that not all physical activities produce equivalent cognitive benefits. Structured sports such as football and table tennis, together with interventions incorporating substantial cognitive demands, including the BARAN and EXCIR programs, produced the most consistent improvements. These activities require continuous decision-making, adaptation to changing environmental conditions, and simultaneous engagement of motor and executive processes, characteristics that are consistent with the superior cognitive benefits reported for open-skill activities [4]. Aquatic exercise should also be considered a promising intervention, as recent evidence identifies it as one of the most effective modalities for improving cognitive flexibility [55]. Likewise, exergaming interventions demonstrated favorable outcomes while also presenting relatively low dropout rates, suggesting greater adherence and motivation compared with more traditional exercise programs [56,57].

The pattern of findings observed across multi-session and acute interventions may be interpreted in light of the neurobiological mechanisms illustrated in Figure 1. Acute exercise is thought to induce transient increases in catecholamines, particularly dopamine and noradrenaline, producing short-term improvements in attention, processing speed, and certain executive functions. In contrast, repeated physical activity over several weeks promotes longer-lasting neuroplastic adaptations through increased brain-derived neurotrophic factor (BDNF) expression, enhanced synaptic plasticity, improved cerebral blood flow, and structural and functional adaptations within the prefrontal cortex and basal ganglia. These cumulative changes provide a plausible explanation for the more robust and sustained improvements observed following chronic interventions.

Another potential mechanism underlying these improvements is the beneficial effect of physical activity on sleep. Sleep disturbances, particularly prolonged sleep latency, are highly prevalent in children with ADHD and are closely associated with impairments in executive functioning [45]. In the studies reporting mediation analyses [45,50], reductions in sleep latency were associated with improvements in cognitive flexibility, suggesting that sleep regulation may partially mediate the cognitive benefits of exercise. Improved sleep may facilitate executive functioning through enhanced memory consolidation, synaptic plasticity, and restoration of prefrontal cortical activity. Nevertheless, none of the included studies formally controlled for sleep as a mediating variable, highlighting the exercise–sleep–cognitive flexibility pathway as an important avenue for future research.

Among the strengths of this review are the comprehensive search strategy across six major electronic databases, the application of design-appropriate critical appraisal and reporting guidelines (PEDro, JBI, and CARE), and the inclusion of 634 participants from 17 studies conducted across multiple countries. Furthermore, by focusing exclusively on children aged 6–12 years, the review provides conclusions that are specifically applicable to the developmental stage of Primary Education.

An important consideration when interpreting the findings is the heterogeneity of both the assessment instruments and the methodological quality of the included studies. Although all selected studies evaluated executive processes related to cognitive flexibility, the assessment tools measured different cognitive constructs. For example, the Wisconsin Card Sorting Test primarily evaluates concept formation, rule shifting, and perseverative responding, whereas the Trail Making Test Part B mainly assesses set-shifting, divided attention, and processing speed. Likewise, the Flanker Task combines inhibitory control with switching performance, while the Stroop Test predominantly assesses cognitive inhibition rather than cognitive flexibility itself. Consequently, the reported improvements may reflect enhancements in different components of executive functioning rather than a single, uniform construct of cognitive flexibility, limiting direct comparisons across studies.

Furthermore, the methodological quality of the included studies ranged from low to good according to the PEDro scale. Several studies lacked participant or assessor blinding, allocation concealment, or intention-to-treat analyses, while others included relatively small sample sizes. These methodological limitations may have introduced bias and influenced the magnitude of the reported effects. In addition, some studies reported exceptionally large effect sizes [47,48]. Although these findings suggest substantial benefits of certain interventions, effect sizes of this magnitude are uncommon in sport and exercise science and may be overestimated in relatively small samples. Consequently, these results should be interpreted cautiously until replicated in larger, well-powered randomized controlled trials. Overall, greater confidence should be placed in studies of moderate-to-good methodological quality, and future research should employ standardized cognitive assessments together with rigorous methodological designs to strengthen the current evidence and facilitate comparisons across intervention studies.

### 4.1. Limitations

However, several limitations should be considered when interpreting the findings of this review. First, there was a marked sex imbalance among participants, with 535 boys and only 99 girls included across the selected studies. This disparity limits the generalizability of the findings to the broader population of children with ADHD, particularly girls, who remain underrepresented in the current literature. Second, substantial methodological heterogeneity was observed across studies, including differences in intervention protocols (type, frequency, duration, and intensity of physical activity), study designs, and outcome measures used to assess cognitive flexibility. This variability precluded direct comparisons between studies and limited the feasibility of quantitative synthesis. Furthermore, eight studies did not report exercise intensity, restricting the ability to establish evidence-based recommendations regarding the optimal dose of physical activity for improving cognitive flexibility. Moreover, psychostimulant medications may influence the same dopaminergic and noradrenergic pathways proposed to mediate the cognitive benefits of physical activity. However, medication use, dosage, and assessment procedures were not consistently reported or controlled, making it impossible to determine the independent contribution of physical activity to the observed improvements in cognitive flexibility. Future studies should systematically report and control for medication status.

An additional limitation is that the review protocol was registered retrospectively in the PROSPERO database rather than prospectively. The review was originally conducted as part of a Master’s thesis, without an initial intention to publish the findings in a scientific journal. Consequently, the protocol was registered only after the review had been completed, when the manuscript was being prepared for publication. Although retrospective registration may theoretically increase the risk of selective outcome reporting bias, the eligibility criteria, search strategy, outcome measures, and methodological approach remained unchanged throughout the review process. Therefore, we consider that retrospective registration did not influence the conduct or conclusions of the review; nevertheless, this should be taken into account when interpreting the findings.

Finally, the possibility of publication bias cannot be excluded. Studies reporting positive effects of physical activity interventions are generally more likely to be published than those reporting null or negative findings, potentially leading to an overestimation of the overall benefits observed in the literature. Because no meta-analysis was performed and the included studies were highly heterogeneous in terms of design, interventions, and outcome measures, formal statistical assessment of publication bias (e.g., funnel plots or Egger’s regression test) was not appropriate. Consequently, the results should be interpreted with caution, acknowledging that unpublished studies may influence the overall strength of the available evidence.

### 4.2. Future Perspective

For Physical Education teaching, these findings suggest that programming should prioritize varied, playful activities with high perceptual demand over purely cyclical exercise, provided that the objective is to enhance cognitive flexibility in students with ADHD. Integrating dual tasks (motor and cognitive) and team sports may represent a promising strategy for this type of student. For future research, it is essential to increase the representation of girls in samples and to conduct studies that directly compare different types of exercise under controlled intensity protocols. At the same time, it would be valuable to further explore the mediating role of variables such as sleep or intrinsic motivation in the improvement of executive functions. In conclusion, although the evidence is promising, more homogeneous studies are required to establish more precise guidelines for the prescription of physical activity for students with ADHD in the context of Primary Education.

## 5. Conclusions

The available evidence suggests that regular physical activity can improve cognitive flexibility in Primary Education students with ADHD. Interventions incorporating cognitive and coordinative demands, such as structured sports and cognitively engaging exercise programs, appear to produce the most consistent benefits in executive functioning. Moreover, multi-session interventions tended to report more consistent improvements than single-session protocols, although the heterogeneity of the available evidence precludes conclusions regarding their comparative superiority.

## Figures and Tables

**Table 1:** Characteristics of the studies included in the review.

First Autor, Year	Country	Study Design	Participant Characteristics				Intervention					
			Total	M	F	Mean Age (Years)	Type	Intensity	Duration (min)	Frequency (Times/Week)	Time (Weeks)	Measurement Instrument
Barudin-Carreiro, 2024 [38]	USA	RCT	22	15	7	9.5	Walking, standing and sitting		20	1	1	WCST
Benzing, 2018 [39]	Switzerland	RCT	46	38	8	10.5	Exergaming	Moderate to vigorous	15	1	1	Flanker Task
Benzing, 2019 [40]	Switzerland	RCT	51	42	9	10.6	Exergaming	Moderate to vigorous	30	3	8	Flanker Task
Bigelow, 2021 [52]	Canada	Within-Subject	16	11	5	11.4	Aerobic exercise and mindfulness	Moderate	10	1	1	TMT
Cabala-Olazábal, 2024 [54]	Peru	Case Report	4	2	2	7	Body rhythmic sequences		60	5	5	WCST
Chang, 2012 [14]	Taiwan	RCT	40	37	3	10.4	Aerobic exercise	Moderate	30	1	1	WCST
Silva, 2019 [41]	Brasil	RCT	20	14	6	12	Swimming		45	2	8	TMT
Hung, 2016 [42]	Taiwan	RCT	34	33	1	10.2	Aerobic exercise	Moderate	30	1	1	Task-Switching Paradigm
Jun, 2023 [43]	China	RCT	74	74	0	6.8	Football		NR	NR	6	Task-Switching Paradigm via E-Prime 2.0
Kang, 2011 [44]	South Korea	RCT	28	28	0	8.5	Aerobic exercise, goal-directed training and group jump rope		90	2	6	TMT
Liang, 2022 [46]	China	RCT	80	62	18	8.5	Aerobic exercise and neurocognitive exercise	Moderate to vigorous	60	3	12	TMT
Memarmoghaddam, 2016 [47]	Iran	RCT	36	36	0	8.3	Selected exercises	Moderate to high	90	3	8	Stroop Test
Nejati, 2021 [48]	Iran	RCT	29	16	13	9.5	BARAN		45	3	4–5	WCST
Nejati, 2021 [48]	Iran	RCT	30	26	4	9.4	EXCIR		40–50	3	4–5	WCST
Pan, 2015 [50]	Taiwan	RCT	30	30	0	9	Table tennis		70	2	12	WCST
Piepmeier, 2015 [53]	USA	Within-Subject	14	9	5	10.1	Aerobic exercise	Moderate	30	1	1	TMT
Qiu, 2024 [51]	China	RCT	80	62	18	8.3	Aerobic exercise and neurocognitive exercise	Moderate to vigorous	60	3	12	TMT

M = male; F = female. Min = minutes. NR = not reported.

**Table 2:** Critical appraisal of the included studies.

***The Physiotherapy Evidence Database scale (PEDro)***													
**First Author, Year**	**Item 1**	**Item 2**	**Item 3**	**Item 4**	**Item 5**	**Item 6**	**Item 7**	**Item 8**	**Item 9**	**Item 10**	**Item 11**	**Score**	**Study Quality**
Barudin-Carreiro, 2024 [38]	1	1	1	0	0	0	0	1	1	1	1	6	good
Benzing, 2018 [39]	1	1	1	1	0	0	0	1	0	1	1	6	good
Benzing, 2019 [40]	1	1	1	1	0	0	0	1	1	1	1	7	good
Chang, 2012 [14]	1	1	0	1	0	0	0	1	1	1	1	6	good
Silva, 2019 [41]	1	1	0	1	0	0	0	0	0	1	1	4	acceptable
Hung, 2016 [42]	1	0	0	0	0	0	0	1	0	1	1	3	low
Jun, 2023 [43]	1	1	0	1	0	0	0	1	0	1	1	5	acceptable
Kang, 2011 [44]	1	1	0	1	0	0	0	1	0	1	1	5	acceptable
Liang, 2022 [46]	1	1	0	1	0	0	0	1	0	1	1	5	acceptable
Memarmoghaddam, 2016 [47]	1	1	0	1	0	0	0	1	0	1	1	5	acceptable
Nejati, 2021 [48]	1	1	0	1	0	0	0	1	0	1	1	5	acceptable
Nejati, 2021 [49]	1	1	0	1	0	0	0	1	1	1	1	6	good
Pan, 2015 [50]	1	0	0	1	0	0	0	1	1	1	1	5	acceptable
Qiu, 2024 [51]	1	1	0	1	0	0	0	1	0	1	1	5	acceptable
***The Joanna Briggs Institute (JBI) Critical Appraisal Tools***													
**First Author, Year**	**Item 1**	**Item 2**	**Item 3**	**Item 4**	**Item 5**	**Item 6**	**Item 7**	**Item 8**	**Item 9**	**N/A**	**N/A**	**N/A**	**N/A**
Bigelow, 2021 [52]	Yes	Yes	Yes	Yes	Yes	Yes	Yes	Yes	Yes	N/A	N/A	N/A	N/A
Piepmeier, 2015 [53]	Yes	Yes	Yes	Yes	No	Yes	Yes	Yes	Yes	N/A	N/A	N/A	N/A
***Case Report Guidelines (CARE Guidelines)***													
**First Author, Year**	**Item 1**	**Item 2**	**Item 3**	**Item 4**	**Item 5**	**Item 6**	**Item 7**	**Item 8**	**Item 9**	**Item 10**	**Item 11**	**Item 12**	**Item 13**
Cabala-Olazábal, 2024 [54]	No	Yes	Yes	Yes	Yes	Yes	No	Yes	Yes	Yes	Yes	No	Yes

N/A = not applicable.

**Table 3:** Results about cognitive flexibility.

First Author, Year	Intervention	Instrument	Main Findings	Key Effect
Barudin-Carreiro, 2024 [38]	Walking, standing, sitting (acute)	WCST	Standing produced the greatest improvement across all WCST outcomes compared with walking and sitting.	↑ Total errors, perseverative errors and conceptual responses (5–14% improvement).
Benzing, 2018 [39]	Exergaming (acute)	Flanker Task	Improved reaction time and reduced switch costs without affecting accuracy.	η^2^p = 0.11; *p* = 0.024.
Benzing, 2019 [40]	Exergaming	Flanker Task	Faster reaction times after intervention; no significant change in accuracy.	d = 0.65; *p* = 0.029.
Bigelow, 2021 [52]	Aerobic exercise + mindfulness	TMT-B	Mindfulness produced a short-term improvement; acute exercise showed no significant effects.	d = 0.56; *p* = 0.04 (mindfulness only).
Cabala-Olazábal, 2024 [54]	Rhythmic body sequences	WCST	No significant changes in cognitive flexibility.	*p* > 0.05.
Chang, 2012 [14]	Acute aerobic exercise	WCST	Improved non-perseverative errors and completed categories; limited effects on remaining variables.	η^2^p = 0.17–0.34; *p* < 0.05.
Silva, 2019 [41]	Swimming	TMT-B	Significant improvement after swimming program.	65% improvement; *p* = 0.042.
Hung, 2016 [42]	Acute aerobic exercise	Task Switching Paradigm	Faster responses but no improvement in switching cost or accuracy.	No significant intervention effect.
Jun, 2023 [43]	Football	E-Prime	Football improved cognitive flexibility over time.	Time × group interaction: *p* < 0.01.
Kang, 2011 [44]	Sports therapy	TMT-B	Reduced completion time after intervention.	*p* = 0.04.
Liang, 2022 [46]	Combined exercise + neurocognitive training	TMT-B	Improved completion time and reduced errors; benefits associated with reduced sleep latency.	η^2^ = 0.09–0.24; *p* < 0.01.
Memarmoghaddam, 2016 [47]	Selected exercises	Stroop Test	Significant improvements across Stroop outcomes.	η^2^ = 0.86; *p* < 0.001.
Nejati, 2021 [48]	BARAN	WCST	Reduced perseverative errors and increased completed categories; improvements maintained at follow-up.	*p* < 0.001.
Nejati, 2021 [48]	EXCIR	WCST	Large improvements in completed categories and perseverative errors compared with aerobic exercise.	η^2^p = 0.52–0.82; *p* < 0.001.
Pan, 2015 [50]	Table tennis	WCST	Significant improvements in all main WCST variables.	d = 0.78–1.19; *p* < 0.05.
Piepmeier, 2015 [53]	Acute aerobic exercise	TMT-B	No significant effects on cognitive flexibility.	*p* > 0.05.
Qiu, 2024 [51]	Combined exercise + neurocognitive training	TMT-B	Improved cognitive flexibility, partially mediated by reduced sleep latency.	β = −0.30; *p* = 0.01.

Abbreviations: WCST, Wisconsin Card Sorting Test; TMT-B, Trail Making Test Part B; EXCIR, Exercise-Cognitive Intervention Program; BARAN, Balance-based Attentive Rehabilitation of Attention Networks; SD, standard deviation; CI, confidence interval; β, standardized regression coefficient; η^2^, eta squared; η^2^p, partial eta squared; d, Cohen’s *d*; ms, milliseconds; ↑ indicates an increase relative to the reference value.

## Data Availability

All data analyzed in this systematic review are derived from previously published studies, which are cited in the manuscript.

## Supplementary Materials

The following supporting information can be downloaded at: https://www.mdpi.com/article/10.3390/sports14090389/s1, Table S1: The PRISMA 2020 statement: an updated guideline for reporting systematic reviews.

## Abbreviations

The following abbreviations are used in this manuscript:

ADHD	Attention Deficit Hyperactivity Disorder
ANCOVA	Analysis of Covariance
BARAN	Balance-based Attentive Rehabilitation of Attention Networks
BDNF	Brain-Derived Neurotrophic Factor
CARE	Case Report Guidelines
EXCIR	Exercise-Cognitive Intervention Program
JBI	Joanna Briggs Institute
MANCOVA	Multivariate Analysis of Covariance
PEDro	Physiotherapy Evidence Database
PICO	Population, Intervention, Comparison and Outcome
PRISMA	Preferred Reporting Items for Systematic Reviews and Meta-Analyses
RCT	Randomized Controlled Trial
TMT	Trail Making Test
WCST	Wisconsin Card Sorting Test
